# The relationship between elevated plasma zonulin levels and Hashimoto’s thyroiditis

**DOI:** 10.55730/1300-0144.5352

**Published:** 2021-12-09

**Authors:** Esra DEMİR, Burak ÖNAL, Hanişe ÖZKAN, İrem Kıraç UTKU, Berrak ŞAHTİYANCI, Abdülbaki KUMBASAR, Güven YENMİŞ, Bülent DEMİR

**Affiliations:** 1 Department of Internal Medicine, Faculty of Medicine, İstanbul Medipol University, İstanbul, Turkey; 2 Department of Medical Pharmacology, Faculty of Medicine, Biruni University, İstanbul, Turkey; 3 Department of Internal Medicine, Kanuni Sultan Süleyman Training and Research Hospital, Health Sciences University, İstanbul, Turkey; 4 Department of Medical Biology, Faculty of Medicine, Biruni University, İstanbul, Turkey; 5 Department of Cardiology, Turkish Hospital, Doha, Qatar

**Keywords:** Hashimoto thyroiditis, autoimmune diseases, microbiota, gut permeability, zonulin

## Abstract

**Background/aim:**

Hashimoto thyroiditis (HT) is one of the most prevalent autoimmune diseases. The intestine microbiota is strongly associated with autoimmune diseases. Zonulin, a modulator of tight junctions that controls the selective permeability of the intestine can induce an elevation in gut permeability. We aimed to investigate the association of plasma zonulin levels with HT.

**Materials and methods:**

We compared 77 HT patients with 66 age-gender and BMI-matched healthy individuals in the case of plasma zonulin levels. Plasma zonulin levels were measured by ELISA. The statistical analyses were performed using Student’s t-test and chi-square tests. The predictive power was investigated using univariate and multivariate logistic regression analysis.

**Results:**

We found that the increase in plasma zonulin levels in the HT group was statistically significant compared to the control group (p < 0.001). The regression analysis showed that urea, anti-thyroid peroxidase, aspartate aminotransferase, thyroid-stimulating hormone, free T3, and serum zonulin levels were found to be associated with HT in both univariate and multivariate models (p < 0.05).

**Conclusion:**

Zonulin is a possible biomarker candidate that may link intestinal permeability with the etiology of autoimmune diseases.

## 1. Introduction

The first serious description and analyses of autoimmune thyroiditis leading to primary hypothyroidism emerged during the 1910s [1]. Hakaru Hashimoto, in 1912, defined a distinctive hypothyroidism disease in which inflammatory cells infiltrate into thyroid tissue resulting in the production of antibodies against thyroid hormones such as triiodothyronine (T3) and thyroxine (T4) which are synthesized by a pivotal enzyme, namely thyroperoxidase (TPO) [2]. Generally speaking, commonly observed in females (>15% of +60 years females but <2% of males) [3], this disease is a major public health problem which corresponds to 10% of all adult hypothyroiditis patients [4] and is called “Hashimoto’s thyroiditis (HT)” after being discovered. The disease displays its symptoms via the elevation of antibodies (anti-TPO) against enzyme TPO which is observed in nearly 20% of general society and almost always increases in HT [5].

The intestinal epithelium, composed of single-layered epithelial cells lining the small intestine, provides the largest mucosal barrier between the external environment and the human host [6]. The selectively regulated passage of macromolecules is maintained by intestinal paracellular permeability which depends on the integrity of one of the hallmarks of absorptive and secretory epithelia, intercellular tight junctions (TJs) [7]. TJs are highly dynamic structures capable of rapid and coordinated responses that separate the basolateral and apical cellular compartments. In healthy conditions, quantitively small macromolecules with active immunogenicity are allowed to cross the mucosal barrier [8].

The intestinal microbiota displays a substantial function to maintain the homeostasis of the gastrointestinal system and may be at the heart of our understanding of thyroid disorders [9]. Intestinal flora teeming with various microbial organisms may explain the large differences among different world regions in the prevalence of goiter, a thyroid disorder mainly caused by insufficient iodine (I−) intake. For example, in southern India, goiter is not associated with I-intake, whereas hypothyroidism is more common in iodine-rich areas of Japan than in iodine-poor areas [10,11].

The end products of intestinal microbiota such as short-chain fatty acids are reservoirs of energy to be served in the production of thyroid hormones and strengthening intercellular TJs [12]. The cooperation of intestinal content and intercellular TJs stabilizes homeostasis in case of the immune response to nonself antigens thus the presence of an aberrant intestinal gut microbiome triggers the proliferation of auto-reactive T-cells [13].

Considering the possible external inducers of HT, the gut emerges as a key region, as the immune system with mucosa experiences and responds to the outside environment for the first time. Many autoimmune diseases such as diabetes, inflammatory bowel disease (IBD), and neurodegenerative diseases, cardiovascular diseases and cancers are being associated with microbiota [14], while several ones with the aberrant intestinal barrier permeability [15].

**Figure 1:**
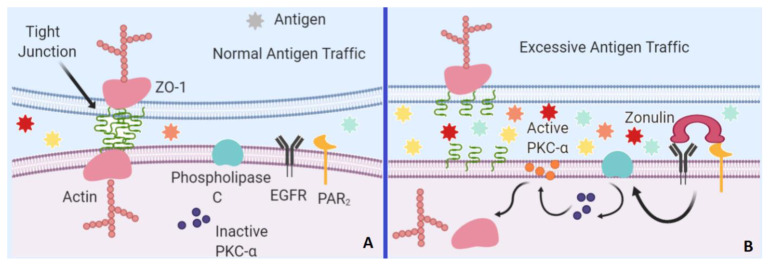
ZO-1 is the main part of the TJs. A. During normal antigen trafficking, ZO-1 is strictly bound to TJs complex with the help of actin filaments leading to selectively permeability of the intestinal barrier. **B**. In the presence of zonulin, the activated EGFR and PAR_2_ receptors trigger phospholipase C activation which in turn activates PKC-α. Activated PKC-α then triggers the release of ZO-1 and actin filaments leading to dysfunction of TJs and aberrant barrier permeability. PAR_2_: protease-activated receptor 2, PKC-α: Protein Kinase C Alpha, ZO-1: Zona Occludens 1, EGFR: Epidermal growth factor receptor (the figure was drawn using biorender.com).

Zonulin is a paracrine signaling protein largely secreted by small intestine epithelial cells that coordinate the opening of intestinal TJs [16]. In normal antigenic trafficking within the small intestine, TJs’ main component, zonula occludens 1 (ZO-1) form the TJs complex with the help of actin filaments binding. However, zonulin binding to two transmembrane receptors, namely protease-activated receptor 2 and epidermal growth factor receptor triggers a cascade in which phospholipase C activates protein kinase C alpha, which in turn catalyzes the phosphorylation of ZO-1 and other target proteins and the depolymerization of actins [17] which eventually results in a loose conformation of TJs, increased permeability, and excessive-abnormal antigenic trafficking (Figure 1).

Based on the relationship between the intestinal microbiome, intestinal permeability, and autoimmunity, we sought to explore whether elevated levels of plasma zonulin contributed to HT. Here, we discuss the crosstalk between plasma zonulin levels and HT susceptibility, which may improve target-specific therapy for HT.

## 2. Materials and methods

### 2.1. Study subjects

The study enrolled newly diagnosed 77 Hashimoto thyroiditis patients (75 of females and 2 of males) and age-gender-BMI matched 66 healthy subjects (65 of females and 1 of male) at the Internal Medicine Department outpatient clinic of Kanuni Sultan Süleyman Traning and Research Hospital, İstanbul, Turkey.

Generally speaking, the individuals with an increased thyroid-stimulating hormone (TSH) and low levels of free thyroxine (FT4), with elevated antithyroid peroxidase (TPO) antibodies demonstration coupled with the physical examination and differential diagnosis from many other thyroid gland related disorders or autoimmune diseases are included within the study as the patients’ group [18]. Individuals with no systemic or autoimmune disease history are included as the control group. The mean age was 36.8 ± 9.3 years in the patients and 34.6 ± 11.4 in control groups. All procedures were performed following the ethical standards of the Institutional Ethics Committee of our hospital (Ethics Committee decision no: KAEK-2019.04.87) and with the 1964 Helsinki Declaration ethical standards. All study subjects were inhabitants of İstanbul having Turkish origin and provided signed informed consent prior to the sample and data collection.

### 2.2. Sample preparation

After the patient or the control had fasted for at least 8 h, peripheral blood samples were taken from antecubital veins in the morning, thereafter, samples were collected in tubes with EDTA for biochemical analysis and without additives for serum zonulin level. The following parameters were run simultaneously. In biochemical analysis, complete blood count, liver function parameters such as gamma-glutamyltransferase (GGT), alanine aminotransferase (ALT) and aspartate aminotransferase (AST), potassium and sodium levels, fasting blood glucose, HbA1c, thyroid-stimulating hormone, free T3, free T4, anti-thyroid peroxidase, triglyceride, high-density lipoprotein cholesterol (HDL), low-density lipoprotein cholesterol (LDL), total cholesterol, urea, uric acid, and creatinine were measured. An Abbot Aeroset 2.0 (Abbot Diagnostic, USA) was used for spectrophotometric calculation of the amount of liver enzymes ALT, AST, and GGT, lipid profiles, uric acid and glucose levels, whereas Sysmex XN 9000 hematology analyzer (Symex Europe GmbH, Germany) was for the complete blood count, Cobas 8000 C702 (Roche Diagnostics, USA) chemistry analyzer was for Na and K electrolytes and Variant 2 Turbo (Biorad, USA) was for HbA1c%. Plasma zonulin concentrations were determined by an enzyme-linked immunosorbent assay (Human Zonulin ELISA kit, Cat no: E3704Hu, Bioassay Technology Laboratory, Shanghai, CHINA). The intra-assay precision of the kit is <8%, whereas the interassay precision is <10%. The precision (%) is calculated by the equation of SD/mean × 100.

### 2.3. Statistical analysis

In the descriptive statistics of the data, the median, standard deviation, average, frequency, lowest-highest values, and ratio were determined. The distribution of variables was measured by the Kolmogorov Smirnov test. In the analysis of quantitative independent data, normal distribution between groups was evaluated using Student’s t-test, while the Mann–Whitney U test was used for nonnormally distributed data. The qualitative independent data were evaluated by the chi-square test. In the cases the chi-square test conditions were not met, the Fischer test was applied. The predictive power was investigated using univariate and multivariate logistic regression analysis. The predictive power and cut-off value were analyzed with the ROC curve. The Statistical Package for Social Sciences statistical software release 22 (IBM Corporation, Armonk, NY) was used for the statistical calculations.

## 3. Results

The demographics, laboratory parameters and clinical features of both control and HT patients are summarized in Table 1 and Table 2.

There was no significant difference between the Hashimoto thyroiditis and control groups concerning age, gender, height, weight, and body mass index (p > 0.05) for all comparisons (Table 1).

**Figure 2:**
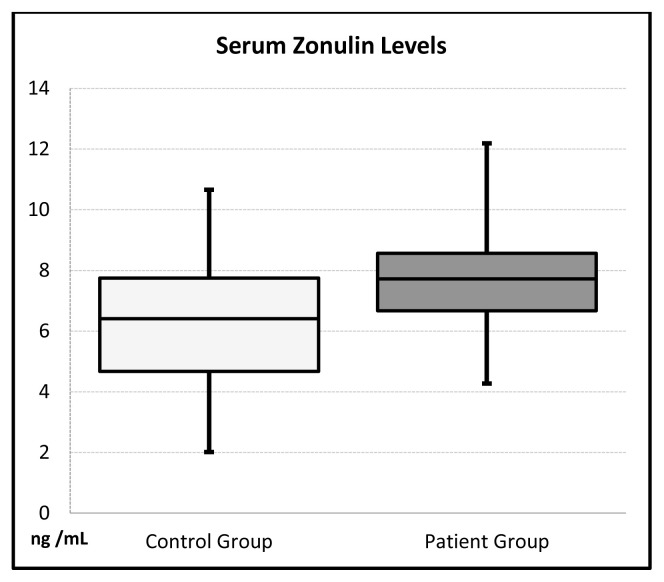
The comparison of plasma zonulin levels between control and patient groups.

The fasting glucose, total cholesterol, LDL, HDL, uric acid, creatinine, TG, HgA1c, ALT, GGT, Na, K, FT4, WBC, PLT, MPV, and Hgb levels were not significant statistically between the patient and control groups. The level of ure (p = 0.010), anti-TPO (p ≤ 0.001), AST (p = 0.005), TSH (p ≤ 0.001), and serum zonulin (p≤0.001) (Figure 2) elevated significantly whereas the FT3 level decreased (p = 0.008) in patient groups compared to control group (Table 2).

Uni/multivariate regression analysis was performed to determine the possible influence of urea, anti-TPO, AST, TSH, FT3, and serum zonulin on the presence of HT. Urea, anti-TPO, AST, TSH, FT3, tri-iodothyronine, and serum zonulin levels were found to be associated with HT in both univariate and multivariate models (p < 0.05) (Table 3).

**Figure 3:**
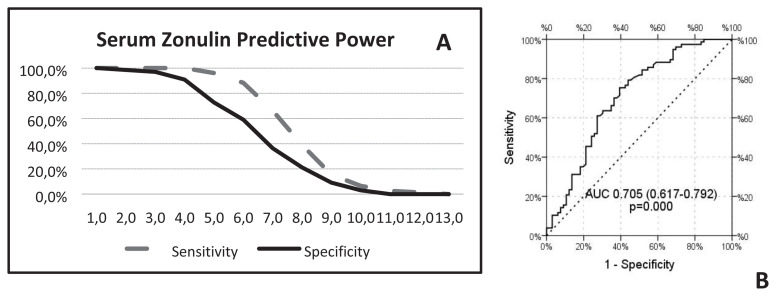
A**-**The diagrammatic illustration of serum zonulin predictive power in case of sensitivity and specificity. B**-**The efficiency of plasma zonulin levels is found statistically significant [area under the curve 0.705 (0.617–0.792)].

To determine the predictive power of plasma zonulin levels in the case of HT, a ROC analysis was performed (Figure 3-A,B) (Table 4). The plasma zonulin level of ≥4 ng/mL had a sensitivity (100%) so the positive predictive value (PPV) of 100% whereas a specificity (90.6%) so the negative predictive value (NPV) of 90.9% (Table 4). In the discrimination of the patient and control group, we observed a statistically significant efficiency of plasma zonulin levels [area under the curve 0.705 (0.617–0.792)] (Figure 3-B).

## 4. Discussion

The most important result of our study is that plasma zonulin levels were higher in the HT group than in the control group. In this context, zonulin as a marker of impaired intestinal permeability may play a role in the pathogenesis of autoimmune diseases.

Humans come to earth from a sterile nature fulfilled with amniotic fluids and afterward the surfaces layered by epithelia such as skin, genital, nasal, and respiratory system tracts are covered systemically by microorganisms [19]. For human survival, it is mandatory to maintain tissue homeostasis. The homeostasis is balanced by the interaction between the host and the microorganisms living inside it. This is the code for a multicellular organism’s existence as both the symbiotic commensal microbiota inside the host and the macroscopic host are the two sides of the same coin. This substantial task is succeeded by a complex but coordinated set of both adaptive and innate reactions that modulate responses optimally against nutrients, commensal microorganisms, and probable pathogens [20]. Although the breakthrough in understanding the role of microbiota in the gut was Do derlein’s work which reported a specific bacteria type as the gate-keeper of the vaginal ecosystem in 1892, more than two thousand years ago, Hippocrates the father of medicine, claimed that all disease begins in the gut.

Autoimmune disorders are described as self-tissue damage presence in diverse regions of the human body. Innate cells, circulating autoantibodies, and eventually autoreactive lymphocytes are the three arms of the inflammation, however, the mechanisms responsible for the triggering and maintaining the process remain to be figured out completely. Gut commensal microorganisms as being the target of the adaptive immune system display a connective role in homeostasis [21]. Thus, any alteration in the population of these microorganisms is directly connected to autoimmune diseases. Generally speaking, patients having autoimmunity disorders represent proof of aberrant gut barriers which in turn lead to an immune response to commensal gut bacteria [7].

There is a growing body of evidence regarding the microbiota and its possible role in the autoimmune response. As reported in the Mu survey, a significant alteration in gut microbiota is found between susceptible, healthy or resistant samples in the case of collagen-induced arthritis murine model [22]. Several autoimmune diseases such as systemic lupus erythematosus [23], inflammatory bowel disease [24], systemic sclerosis [25], rheumatoid arthritis [26], type 1 diabetes (T1D) [27] and spondyloarthritis [28] are linked to gut microbiota alteration.

Zonulin, as the key to forcing the intestinal barrier to be more permeable, manages TJs regulation so the reciprocal movement of macromolecules, fluid, and the leukocytes [15].

This research examines the emerging role of the plasma zonulin levels in the context of an autoimmune disease, namely Hashimoto’s thyroiditis. Increased plasma zonulin levels may allow pathogenic antigens to pass the intestinal gut barrier loosening the TJs which can explain the auto-response of innate immunity. Our findings are in agreement with other autoimmune diseases, as plasma zonulin concentrations elevated in celiac disease [17], T1D [29], and inflammatory bowel disease such as Crohn’s disease and ulcerative colitis [30].

Without a shadow of doubt, the discovery of zonulin which is the only physiological gut permeability regulator depicted so far has been the breakpoint to understand the role of intestinal permeability in both disease and health. This study was the first to represent the association between HT and plasma zonulin levels.

The zonulin may be used as a marker in the future to predict clinical response in HT. The small sample size, absence of power analysis before the study, and the lack of additional markers that can affect the gut permeability and the nutritional status that can alter plasma zonulin levels measured are the limiting factors of our study. Additional work with larger sample size is needed not only to confirm our results but also to uncover whether alterations in intestinal microbiota in the case of zonulin are the consequences or the causes of autoimmune diseases.

## Figures and Tables

**Table 1:** The comparison of demographic characteristics between Hashimoto’s thyroiditis and control groups.

Parameters		Control group			Thyroiditis group			p
		Mean ± SD/n-%			Mean ± SD/n-%			
**Age, years**		34.6	±	11.4	36.8	±	9.3	0.202
**Gender**	**Female**	65		98.5 %	75		97.4%	1.000
	**Male**	1		1.5 %	2		2.6%	1.000
**Height, m**		1.6	±	0.0	1.6	±	0.1	0.717
**Weight, kg**		66.3	±	13.4	69.1	±	13.6	0.316
**BMI; kg/m** **^2^**		25.0	±	4.9	25.9	±	5.0	0.352

BMI, Body mass index; s.d, Standard deviation; m, Meter; n, Number; m^2^, Square meter; kg, Kilogram

**Table 2:** The comparison of laboratory parameters between Hashimoto’s thyroiditis and control groups.

Parameters	Control group			Thyroiditis group			p
	Mean ± SD Median			Mean ± SD Median			
**Glucose, mg/dL**	88.7	±	8.5	91.2	±	9.5	0.111
**Urea, mg/dL**	22.9	±	7.2 22	27.8	±	24.3 25	***0.010***
**Creatinine, mg/dL**	0.6	±	0.1	0.6	±	0.1	0.367
**AST, U/L**	15.5	±	2.6	18.0	±	7.8	***0.005***
**ALT, U/L**	14.8	±	4.9 14	16.8	±	11.0 14	0.498
**LDL, mg/dL**	92.5	±	28.5	101.4	±	38.5	0.186
**HDL, mg/dL**	59.5	±	14.6	56.4	±	11.5	0.335
**TG, mg/dL**	86.7	±	49.7	92.1	±	44.3	0.371
**T. Chol, mg /dL**	168.3	±	35.0	174.9	±	45.1	0.579
**Uric acid, mg/dL**	3.7	±	0.9	3.8	±	0.9	0.481
**GGT, U/L**	12.9	±	5.2 11	14.8	±	15.7 11	0.887
**Hb, g/dL**	12.6	±	1.3	12.3	±	1.5	0.338
**WBC, (×10** **^3^** **)**	7.2	±	1.9	7.1	±	1.6	0.934
**Platelets (×10** **^9^** **)**	269.5	±	63.4	278.5	±	55.6	0.382
**MPV, fL**	10.8	±	0.9	11.3	±	7.5	0.092
**Sodium, mmol /L**	140.7	±	2.0	140.3	±	1.5	0.381
**Potassium, mmol /L**	4.4	±	0.3	4.5	±	0.4	0.051
**HbA1c, g/dL**	5.2	±	0.4	5.3	±	0.4	0.358
**TSH, uIU/mL**	2.0	±	1.3 1.6	4.9	±	11.3 3.3	≤***0.001***
**FT3, pg/mL**	3.2	±	0.5	3.0	±	0.5	***0.008***
**FT4, ng / dL**	1.3	±	0.7	1.2	±	0.5	0.935
**ANTI-TPO, IU/mL**	11.9	±	5.0	259.5	±	179.0	≤***0.001***
**Zonulin, ng/mL**	6.4	±	1.9	7.7	±	1.5	≤***0.001***

HbA1C, Hemoglobin A1C; TG, Triglyceride; AST, Aspartate aminotransferase; LDL, Low-density lipoprotein; HDL, High-density lipoprotein; T. Chol, Total cholesterol; Hb, Hemoglobin; ALT, Alanine aminotransferase; GGT, Gamma-glutamyl transferase; WBC; White blood counts, MPV; Mean platelets volume; FT4, Free thyroxine; FT3, Free triiodothyronine; TSH, Thyroid stimulating hormone; ANTI-TPO, Anti-thyroid peroxidase; mg, Milligram; U, Unit; s.d, Standard deviation; L, Liter; n, Number; dL, Deciliter; g, Gram; fL, Femtoliter; mmol, Milimol; mL, Milliliter; pg, Picogram; ng, Nanogram.

**Table 3:** The regression analysis of univariate and multivariate models within the patient group.

	Univariate model					Multivariate model				
	OR	95% CI			p	OR	95% CI			p
Urea	1.05	1.01	-	1.10	***0.047***					
Anti-TPO	1.49	1.06	-	2.08	***0.022***	1.21	1.07	-	1.36	***0.002***
AST	1.16	1.04	-	1.29	***0.007***	1.21	1.07	-	1.36	***0.002***
TSH	1.62	1.29	-	2.04	≤***0.001***	1.62	1.25	-	2.11	≤ ***0.001***
FT3	0.42	0.21	-	0.86	***0.017***	0.35	0.15	-	0.81	***0.015***
Serum Zonulin	1.58	1.26	-	1.98	≤ ***0.001***	1.55	1.21	-	2.00	≤ ***0.001***
OR: Odds ratio, Cl: Confidence interval										

ANTI-TPO, Anti–thyroid peroxidase; AST, Aspartate aminotransferase; TSH, Thyroid stimulating hormone; FT3, Free triiodothyronine

**Table 4:** The sensitivity and specificity of different cut-off values of plasma zonulin levels.

Zonulin	Sensitivity	Specifity
1.0	100.0%	100.0%
2.0	100.0%	98.5%
3.0	100.0%	97.0%
4.0	100.0%	90.9%
5.0	96.1%	72.7%
6.0	88.3%	59.1%
7.0	66.2%	36.4%
8.0	39.0%	21.2%
9.0	15.6%	9.1%
10.0	6.5%	3.0%
11.0	2.6%	0.0%
12.0	1.3%	0.0%
13.0	0.0%	0.0%
